# Demanded interdisciplinary subjects for integration in medical education program from the point of view of graduated medical physicians and senior medical students: a nationwide mixed qualitative-quantitative study from Iran

**DOI:** 10.1186/s12909-024-05079-w

**Published:** 2024-02-07

**Authors:** Kamran Bagheri Lankarani, Behnam Honarvar, Seyed Aliakbar Faghihi, Mohammad Reza Rahmanian Haghighi, Ahmad Kalateh Sadati, Fatemeh Rafiei, Sayyed Amirreza Hosseini, Amir-Hassan Bordbari, Arash Ziaee, Mohammad Jafar Pooriesa

**Affiliations:** 1 Health Policy Research Center, Institute of Health, Shiraz University of Medical Sciences, Shiraz, Iran; 2 Clinical Education Research Center, Shiraz University of Medical Sciences, Shiraz, Iran; 3 Erasmus JMD program. Unit for Research in Emergency and Disaster, University of Oviedo, Oviedo, Spain; 4 Department of Social Sciences, Yazd University, Yazd, Iran; 5 Student Research Committee, School of Medicine, Mazandaran University of Medical Sciences, Sari, Iran; 6 Student Research Committee, School of Medicine, Mashhad University of Medical Sciences, Razavi Khorasan, Iran

**Keywords:** Interdisciplinary, Integration, Medical, Education, Humanities

## Abstract

**Introduction:**

This study aimed to detect interdisciplinary subjects for integration into the medical education program of Iran.

**Methods:**

A qualitative-quantitative method was used. Firstly, interdisciplinary subjects demanded by medical graduates and senior medical students were defined by qualitative study. In the second stage, questionnaire was developed which based on the findings of qualitative stage, experts’ opinion and reviewing of the national general guide of professional ethics for medical practitioners. Questionnaire consisted of demographic, occupational and thirteen interdisciplinary items. These items consisted of social determinants of health, social and economic consequences of disease, social prescribing, physicians’ social responsibility, role of gender, racial, ethnic, social and economic issues in approach to patients, role of logic and mathematics in clinical decision-making, philosophy of medicine, maintaining work-life balance, self-anger management, national laws of medicine, religious law in medical practice, health system structure, and teamwork principles. Level and importance of knowledge and self-assessed educational needs were asked about each item. In the third stage, a national online survey was conducted. SPSS 25 was used for statistics.

**Results:**

By content analysis of data in qualitative stage, 36 sub-themes and 7 themes were extracted. In the quantitative part, 3580 subjects from 41 medical universities across Iran participated in this study. 2896 (80.9%) were medical graduates and 684 (19.1%) were senior medical students. Overall, knowledge about interdisciplinary items was low to intermediate, while high to very high knowledge ranged from maximally 38.7% about socioeconomic consequences of disease to minimally 17.2% about social prescribing. Participants gave the most importance to the having knowledge about self-anger management (88.3%), maintaining work-life balance (87.2%) and social determinants of health (85.8%), respectively. However, national laws of medicine (77.6%), maintaining work-life balance (75.4%) and self-anger management (74%) were the first top three educational demands by participants.

**Conclusion:**

This study revealed a low to moderate level of knowledge about interdisciplinary topics among both graduated medical physicians and senior medical students. These groups showed a strong demand and tendency to know and to be educated about these topics. These findings underscore the urgency for educational reforms to meet the interdisciplinary needs of medical professionals in Iran.

## Introduction

Interdisciplinary education (IE) in medical sciences refers to incorporating multiple academic disciplines into the study and practice of medicine. Biological, chemical, psychological, sociological, financial and public health knowledge are all integral parts of healthcare, which requires a holistic approach. Interdisciplinary education aims to develop critical thinking and creative problem-solving skills in medical students and professionals [1]. Interdisciplinary training allows medical professionals to have a better understanding about the socio-environmental factors that affect their patients’ health. This approach also, fosters robust interpersonal relationships and transparent communication, improving rapport with colleagues and enhancing patient care. By centering on interdisciplinary education, negative biases and prejudices could be diminished, thereby elevating the overall quality of healthcare [2].

Inspired by the World Health Organization’s 2010 report; “Framework for Action on Inter-professional Education and Collaborative Practice,” educational strategists have been increasingly urged to weave inter-professional education and collaborative practices into their curricula. Integrating this interdisciplinary approach into medical education equips students and seasoned professionals with a multifaceted set of skills crucial for excelling in the ever-changing healthcare landscape [3]. This report also, states that “*Interprofessional education occurs when two or more professionals learn about, from and with each other to enable effective collaboration and improve health outcomes*” [4]. The Association of the American Medical Colleges (AAMC), recognized the outstanding and undetected role of arts and humanities in preparing physicians for challenges. This association remarked seven recommendations for integration of arts and humanities into medical education to improve education, practice, and well-being of physicians and their learners. The Liaison Committee on Medical Education also established standards for accreditation of medical education programs in the United States and Canada. Through these standards, a medical school encourages applicants of the medical education program to be pre educated about humanities, natural sciences, and social sciences [5].

Based on another study about the learning objectives of evidence-based medicine in Canadian and American medical schools, most of the objectives emphasized knowledge, comprehension, and application rather than higher levels of critical thinking like analysis, synthesis, and evaluation. Humanities study may provide opportunities to engage with critical thinking at higher levels, but medical education does not consistently value these skills [6]. On the other hand, despite the recognized benefits of IE, some studies suggest that empirical data may not support the assumption that medical education research is interdisciplinary. Instead, it appears that the majority of knowledge entering the field comes from the health research domain. Medical education researchers may lack a thorough understanding of literature from other fields, which can hinder their ability to teach disciplinary knowledge to their students and facilitate knowledge communication with scholars outside medical education [1]. A study suggests that slow progress toward integrating humanities into medical education could result from the belief that humanities are unnecessary or that students will not be interested in them. The fact that students cite disinterest as a reason for avoiding mandatory teaching may reinforce the perception that medical humanities are separate from medicine [7].

In Iran, despite its growing importance and the scholarly debates, the field of medical humanities is conspicuously absent in academic dialogue and course offerings within medical universities and while many experts have articulated diverse opinions on the subject, it has not yet secured the scholarly attention or institutional backing [8–10]. This issue may partly stem from the structural changes to Iran’s medical education system in the 1960s. Modeled after Cuba’s system, Iranian medical education was severed from the broader spectrum of higher education, advocating a social and community-focused medical theory. This framework emphasized the interconnectedness of healthcare and education. The approach has several advantages, such as eliminating the need to import physicians from other countries, well-distributed medical education across various geographical locations, and expanding medical research and activities [11]. However, the ministry of health of Iran identified critical weaknesses in its report; titled “Achievements, Challenges, and Future Directions for the Health System of the Islamic Republic of Iran.” Among these weaknesses were the focus on specialization at the expense of a broader education, insular thinking, and a lack of horizontal and vertical curriculum integration. The report also highlighted a significant gap in communication within the country’s medical education system [12]. As another study emphasized, the contents of medical education programs in Iran are generally disease-oriented while healthcare has been neglected. This study concluded that similar to a lot of efforts that have been made in western countries to revise the curriculum of medical students in line with changes, demographic needs and technology progressions, an evolution of medical education is also needed in Iran. This study also resulted that enrichment of medical education with social prescription is the gateway toward strengthening of primary health care [13].

To answer the increasing demands in the field of medicine in new era, a mere integration of basic and clinical sciences is not enough because it is necessary to emphasize the importance of humanism as well as health population sciences in medicine. It is necessary to integrate basic and clinical sciences, humanism, and health population not only in the early years but also throughout the medical education curriculum to making medical students more prepared and effective in facing real situations [14, 15].

Above evidences show that currently in Iran, the development of interdisciplinary cooperation is not of much interest at medical sciences universities [16] and implementation of IE, especially changing of cultural and attitude toward it is a great challenge [17]. Therefore, to highlight the importance of IE and to provide field-based evidences for policymakers and strategists of medical education in Iran, this study aimed to demonstrate some interdisciplinary items which medicine graduates and pre-graduates think that they need to be educated about them and a gap toward them is existed in medical curriculum.

## Methods

**Fig. 1:**
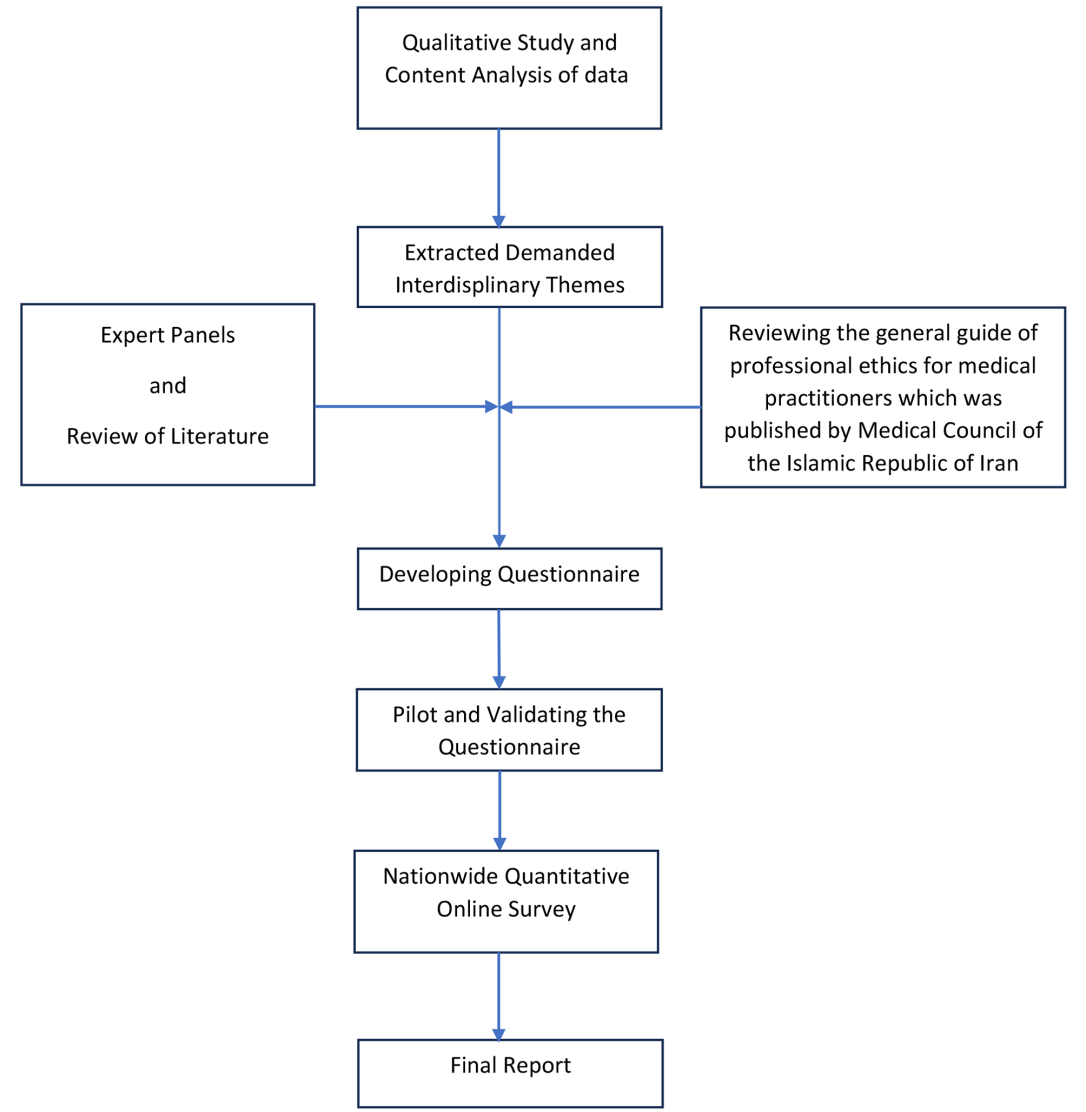
Flowchart of methodology of study

This study was a mixed qualitative and quantitative study that was conducted in 2022 by health policy research center that is affiliated with institute of health in Shiraz University of Medical Sciences (SUMS), Shiraz, Iran. The overall process of study was illustrated in a flowchart (Fig. 1).

### 1-Qualitative study

Stage one of this study was conducted as a qualitative study in five universities including Shiraz, Fasa, Jahrom, Hormozgan and Yasuj medical universities which are located in the south and south-west of Iran. Conceptual content analysis was used to determine the view of graduated medical physicians and senior medical students about needs to integrate medical curriculum with interdisciplinary items such as human sciences. At first, to find the list of main and tracing questions, a warm up interview was done with five individuals of target groups, while answers of these five interviewees were not added to the finally interviewed persons’ answers. The questions which were extracted in this phase were:

- According to your experiences regarding patient care, how do you evaluate the status of doctors and gaps in their education?
- How do cultural and socio-economic issues affect health care?
- What interdisciplinary items, are needed to be integrated in the medical education curriculum of students or in the re-training program of medical graduates?

In order to conduct main interviews, purposeful sampling with maximum diversity was applied, although the sample size was uncertain and the criterion for termination of interviews was information saturation and no hearing further new points. The criteria for maximum diversity were: gender, geographical distribution of the place of employment (for graduates) or education (for students) in the five mentioned universities and years passed from the general medicine graduation (for graduates).

The interviewer was the faculty member of medical sciences education development center (EDC) of Shiraz university of medical sciences who was expert of qualitative studies. In order to carry out this part of the study, the selected samples were contacted first, and after explaining the objectives of the study, if they agreed, the time and place for the face-to-face interview was determined. Data collection was done using in-depth semi-structured interviews and note-taking. The focus in the interviews was: what are the interdisciplinary educational needs (with focusing on the human science) in the medical education program?

For validation and analysis of answers, following steps were done:

(1) Transcribing interviews and observations (2) Reading the texts line by line and separating the quotes from the content (3) Dividing texts into smaller units of analysis (so that they can be reviewed) (4) Determining meaningful sentences and paragraphs as the unit of analysis (5) Assign a code to each meaningful word, sentence and paragraph (6) Group similar codes together (7) Comparing different codes in terms of similarities and differences within classes (8) Validate the codes and classes by re-reviewing the transcripts (9) Determining the relationship between classes in order to summarize and abstract the data (10) Obtaining the final sub-themes and themes. In order to analyze the data, MAXQDA10 software was used to facilitate the classification of codes. To ensure the accuracy and reliability of the data, the Goba and Lincoln criteria of credibility, confirmability, dependability and transferability were used as scientific accuracy criteria in qualitative research. Acceptability of the data was assessed by an expert team in a qualitative study to check the concepts obtained. In order to accept the data, the researcher had a long-term interaction with the data during two years. The reliability of the data was evaluated by performing peer check or external review (Member Check). The primary external findings of the study were presented to some of the participants in the form of codes and classes, and their opinions were received. Some parts of the interview were analyzed by colleagues who were not present in the study, and based on their analysis, the findings were confirmed. In addition, the findings were repeatedly evaluated by the supervisors and consultants (Expert Check). The evidence of other studies and the opinions and ideas of other researchers and the documentation of the study findings helped to confirm the data. Finally, by providing description of the: concepts, participants, content, data collection and analysis, the methods used and the limitations of the study, the transferability of the data can help other researchers to use and follow this research processes.

### 2-Development of questionnaire

For conducting quantitative study, firstly, we aimed to develop a questionnaire. Therefore, we obtained evidences from the mentioned qualitative stage and several meetings with experts from different fields including basic sciences, community medicine, human science, sociology, psychology and ethics, as well as reviewing the general guide of professional ethics for medical practitioners affiliated with Medical Council of the Islamic Republic of Iran (version 2018). In the next step, a draft of questionnaire was designed to implement the pilot study. Concurrency of conducting this study with COVID-19 pandemic and the impossibility of face-to-face interview, caused that the online version of questionnaire was designed in Porsline digital platform. Then, for conducting pilot study, 80 medical faculty members and senior medical students were selected by systematic random sampling from the list of faculty members and medical students of five medical universities which were participated in the 1st stage of this study. Afterward, they were contacted and after explaining the aims of this stage, link of online questionnaire was sent to their WhatsApp channel. Forty-five (%56) were responsive and completed the questionnaires, including 19 faculty members and 26 medical students. The content (relevancy, clarity, simplicity, and necessity) and face validity of questionnaire were confirmed by experts and its reliability was appropriate according to the calculated Cronbach’s alpha (0.67). The final questionnaire consisted of demographic, disciplinary and occupational related questions and also thirteen interdisciplinary items. These items consisted of social determinants of health, social and economic consequences of disease, social prescribing, physicians’ social responsibility in accidents and disasters, role of gender, racial, ethnic, social, economic, and political status of the patients in approach of physicians to them, role of logic and mathematics in clinical decision-making, philosophy of medicine, maintaining work-life balance, self-anger management, national laws of medicine (medical system regulations, medical judicial laws, etc.), religious (Sharia) law in medical practice, health system structure, and management and teamwork principles. Three aspects which were asked about each of these items were the level of knowledge, importance of knowledge and self-assessed educational needs. Scoring of each answer was done by a 6-point Likert scale from none to very high.

### 3-Quantitative study

In the third stage, a national survey was conducted in two phases. The first phase was conducted through an online distribution of questionnaires. The statistical populations of this phase were medical faculty members (around 10,000) as well as senior medical students (around 9000). All medical universities across Iran were included in this phase. Then a focal point in each university was selected as moderator and after online education, we provided them the last updated list of medical faculty members and senior medical students. Sample size for each of the mentioned subgroups was estimated as 1514 using Cochran’s formula, considering 56% responding rate in the pilot stage, confidence interval 95%, error 5%, both gender, and design effect 2. Subsequently, by a proportional systematic random sampling, needed sample size in each university was defined. To send the questionnaire, at first, interviewees were contacted by phone and after explaining about the study and obtaining their informed consent, questionnaire’s link was sent to their WhatsApp channel. After 2–3 weeks, a reminder short message (SMS) was also sent for them to fill the questionnaire, if they did not fill it before. In the second phase, the target statistical population were graduated medical specialists and subspecialists who were not non-faculty members (around 50,000) and general practitioners (around 90,000). In this phase, correspondence was made with the Medical Council of the Islamic Republic of Iran and subsequently a short message (SMS) text including an introduction about the aims of study and the questionnaire’s link was sent by them to 1514 systematically randomized selected individuals of each subgroup.

### 4-Data analysis in quantitative stage

Chi-squared test in IBM SPSS Statistics 25 was used for comparison of four studied subgroups regarding their interdisciplinary needs. *P* value ≤ 0.05 was considered as the significance level.

### Ethical considerations

In the qualitative stage of this study and in order to comply with ethical points, before collecting the data, the objectives of the research were explained to the participants, and after obtaining their informed consent, interviews were recorded. The interviews were conducted individually in the places suggested by the participants. Also, the freedom of the participants to participate in the research or leave it and the confidentiality of the information was respected, while emphasizing that after the research results are printed and rechecked, the audio files will be deleted. In the quantitative stage, interviewees were contacted by phone or a SMS text was sent for them and after explaining about the study and obtaining their informed consent, questionnaire’s link was sent to them. All filled questionnaires were anonymous and encoded. The protocol of this study considered the 2013 revised Helsinki declaration and it was approved by Iran’s National Strategic Research Center of Medical Sciences by registration number “971928”.

## Results

### Findings of qualitative study

In the qualitative stage of this study, 25 individuals including 17 faculty members and 8 senior medical students were interviewed. Each interview was lasted around 1 h. Open codes included need to education about: biopsychosocial approach to the patients, patient-centered instead of disease-centered approach, management of crisis, stress, anger and time, physicians’ self-care and appropriate communication and respect toward coworkers and patients. They also demanded to be trained about body language, team work, responsibility and responsiveness, health economics, life skills, emotional intelligence, professional ethics, and legal rules and knowledge about culture, tradition and customs of different regional populations. Furthermore, they mentioned a gap in their knowledge about health system of Iran and other countries, interdisciplinary items such as artificial intelligence and telemedicine, social and job security, field research, knowledge translation, social and economic determinants of health, corruption and conflict of interest in health services, spirituality, justice in health, personalized medicine, critical thinking and syndromic approach to diseases. They also requested to enrich medical curriculum with role of the art, humanities, literature and aesthetic in medicine, priority of prevention to treatment in medicine, effect of socioeconomic and political issues on medical physicians’ life and effect of climate change on the health. They also mentioned about depoliticization and meritocracy in the health system. By content analysis of data, 36 sub-themes and 7 themes were extracted (Table 1).

**Table 1:** Themes and sub-themes which were extracted in the qualitative study about needed interdisciplinary items in medical curriculum from the point of view of graduated medical physicians and senior medical students

Theme	Sub-theme
Doctor-Patient interaction	Communication Skills Counseling skills
Medical Culture	Respect Professional Behavior and ethics Personal values of the doctor Social accountability Responsiveness Patient Support Team work Personalized Medicine Justice Avoiding corruption
Socio-Cultural Issues	Social differences Cultural competence
Management	Time Crisis Anger and conflicts Depoliticization of health system
Self-care	Balancing between the personal and professional life
Interdisciplinary items	Health economics Humanities Art Aesthetic Literature Technologies Alternative and complementary medicine Critical thinking Philosophy of medicine Spirituality Climate
Legal issues	Judiciary Insurance Tax Forensic medicine Job security Social security

Examples of Themes-related Quotes:

### Doctor-patient interaction

“*The professional interaction of doctors with patients and clients is neither included in the textbooks nor seen in the behavior of professors and residents, and this it has caused a vicious cycle of wrong interactions and psychological challenges*”.

“*Psychological issues and correct behavioral interaction between the patient and the doctor are neglected in the current education of medical students*”.

The current educational curriculum needs to a fundamental revision. None of the necessary training for managing interpersonal relationships, communication with clients, how to teach the patients and correct sympathy and empathy with them are taught to the graduates.

“*We need to be trained about empathy with patients, how to tell the bad news to patients and management and expression techniques in short dealings with patients”.*

“*The most important factor for the success of doctors, is their attentions and spending of energy in prevention and educating each patient face to face. This has a significant effect on the health of society and reducing unnecessary costs*”.

“*…. In dealing with diseases, the approach should be patient-centered instead of disease-centered*”.

“*…. Appropriate relationship with patients is needed to be educated for all medical students*”.

### Medical culture

“*We also need to be educated about assertiveness skills, problem solving skills and self-esteem skills*”.

“*The issue of self-esteem in medical students is unfortunately neglected by professors and universities*”.

### Socio-cultural-economic issues

“*The effect of social variables such as social capital, social security, social vitality, quality of life and life satisfaction on the social health is not ignorable and these issues need to be educated*”.

“*Assessing the patient’s economic and social financial situation and handling these problems and making the right decision according to the patients’ condition and crisis in providing the best relevant services for the treatment of them are effective*”.

“*The familiarity and mastery of clinical medical students and doctors with the native culture traditions and language of the people and the ability to communicate effectively with them in order to correctly understand and effectively treat their medical problems is necessary*”.

*…None of the necessary training for social issues affecting medicine are taught to the graduates*.

### Management

“*How to control anger has never been taught in the course of general medicine, and this has caused severe challenges in many fields and resulted that the discussion of education has been marginalized*”.

“*Education about stress control and individual anger control in the medical curriculum is necessary*”.

“*We also need to be educated about self-control skills*”.

### Self-care

“*Unfortunately, a useful and effective program to maintain and improve the mental and physical health and vitality of medical students and doctors, who deal with sick and sad patients throughout their studies and practice, has not been taught and this problem affects students and graduates’ morale and health*”.

“*Due to their busy schedules, our doctors have been far away from receiving sufficient education regarding personal life issues. In our society, most of the doctors are one-dimensional people and they usually do not behave properly for themselves*”.

*“How to balance between the personal and professional life, should be educated to medical students* ***”.***

“*How to implement balance between work and family should be trained in the medical curriculum*”.

“*Fun and recreations are necessary for doctors*”.

### Interdisciplinary items

“*Medical education in Iran needs to a complete revisions in all topics*”.

“*In the medical education, we need to a comprehensive approach in recognizing problems and finding solutions in this field. The view in medical education should be with a systemic, preventive and holistic approach, and a health-oriented and proactive approach is needed. The medical education system should be leveled, which means that instead of presenting topics with minute details at once, the general outline of the topics should be presented in a logical and coherent manner, and then this outline should be completed and developed step by step and level by level. It is only in this way that a logical and lasting understanding of the contents with the ability to be used effectively in the long term is formed. In other words, emphasis should be on the mechanisms in recognizing diseases and their diagnosis and treatment instead of emphasizing on memorizing a lot of points about them. The power of inference, critical thinking and problem-based solving education should be developed*”.

“*Personally, I am very interested in interdisciplinary sciences and I am curious about how the disciplines should be related to each other, not that everyone just does his own work. I didn’t see a tutorial for it, I just have raw ideas that I hope can be followed. In addition, I was interested in learning about social psychology, philosophy and medical ethics, and I also had scattered studies, but it was never addressed as it should be in the university environment. We all just follow the same routine, which we are not sure is the right one*”.

“*In my opinion, it is necessary for doctors to use social sciences, history, philosophy and literature more and participate in social affairs such as sports and social associations. They should learn more about the importance of social work and in fact it is necessary to increase their social literacy*”.

“*Knowledge about the basics of health economics and basic economics and the basics of health policy making is necessary*”.

“*Education in the field of personal life skills such as self-awareness, judgment, decision-making, creative thinking and critical thinking is necessary*”.

“*Medical students need to breathe in the academic atmosphere and mingle with professors and students of other disciplines. They should study and discuss various topics and distance themselves from the current one-dimensional education, and freely practice wisdom, democracy and social activities in the university*”.

“*Education about economic aspects of health and healthcare costs is very important in the medical education*”.

Education about climate and its effects on the health is necessary for medical students.

### Legal issues

The current educational curriculum needs to a fundamental revision. None of the necessary trainings for work rules are taught to the graduates.

Doctors’ awareness of non-medical issues (judiciary, forensic medicine, insurance, tax,…) is very low and this is disturbing the practice of medicine.

“*Increasing Knowledge about payment rules, income, expenses and taxes in the public or private system is necessary*”.

“*We need to know about doctors’ pension insurance*”.

### Findings of quantitative study

In the national survey stage of this study, 3580 subjects by mean of age 40.7 ± 12.8 years old and 2020 men (56.4%) and 1560 women (43.6%) from 41 medical universities of all 31 provinces of Iran participated. In term of educational status, 496 (13.9%) were faculty members subspecialists and specialists, 730 (20.4%) were non-faculty members subspecialists and specialists, 44 (1.2%) were fellowships, 238 (6.6%) were residents, 1388 (38.8%) were general practitioners, and 684 (19.1%) were senior medical students. Responding rate was 51.4% (778/1514) in the faculty members, 48.2% (730/1514) in the medical students, 91.7% (1388/1514) in the non-faculty members and 45.2% (684/1514) in the general physicians. The overall responding rate was 59% (3580/ 6056). The mean age of participants was 40.7 ± 12.8 years old, with minimum 24, maximum 92, and median age 40 years old. Considering the year of graduation (in the graduates) from the medical university, 25% had graduated before 1997, 50% before 2008 and 75% before 2015. In terms of academic ranking, 957 (26.7%) claimed that they were belonged to the top 10% of their classmates, 1315 (36.7%) were among 10 to 25% of their classmates, and 1308 (36.5%) were among middle rankings of their classes. Overall, knowledge about interdisciplinary items was low to intermediate, while high to very high level of knowledge ranged from maximally 38.7% about social and economic consequences of disease to minimally 17.2% about social prescribing (Table 2). Considering the Importance of Knowledge about interdisciplinary items, participants rated self-anger management (88.3%), maintaining work-life balance (87.2%) and social determinants of health (85.8%) as the top three important items, respectively, while knowing about religious law in medical practice (41.5%) and role of logic and mathematics in clinical decision-making (44.4%) were ranked as the least important items, respectively (Table 3). In overall, education about national laws of medicine (77.6%), maintaining work-life balance (75.4%) and self-anger management (74%) were as the first top three self-assessed educational needs by participants in this study (Table 4). In term of 13 studied items, a significant difference was noted regarding demanded needs about social prescribing (*p* = 0.001), national laws of medicine (*p* = 0.02), physicians’ social responsibility in accidents and disasters (*p* = 0.02) and religious law in medical practice (*p* = 0.05) among four studied subgroups (Table 5). Furthermore, most of faculty members claimed that they need to be educated about maintaining work-life balance (74.5%), while need to be educated about national laws of medicine was the most demanded item for education in non-faculty members sub-specialists and specialists (76.5%), non-faculty members general practitioners (78.5%) and senior medical students (80.5%) (Table 5). Furthermore, never being educated about 13 studied items was ranged between 42.5% (*n* = 1521) about social determinants of health to 78.7% (*n* = 2819) toward maintaining work-life balance, while a significant difference (*p* < 0.001) was noted among four studied subgroups regarding this question.

**Table 2:** Level of knowledge of medical physicians and medical senior students toward interdisciplinary items (percentage in parentheses)

Interdisciplinary Items	None	Very Little	Little	Medium	High	Very High
Social and economic consequences of diseases	29(0.8)	129(3.6)	332(9.3)	1703(47.6)	1031(28.8)	356(9.9)
Role of gender, racial, ethnic, social, economic, and political status of the patients in providing services to them	67(1.9)	173(4.8)	421(11.8)	1539(43)	892(24.9)	488(13.6)
Self-anger management	132(3.7)	258(7.2)	415(11.6)	1441(40.3)	911(25.4)	423(11.8)
Social determinants of health	27(0.8)	113 (3.2)	345(9.6)	1780(49.7)	956 (26.7)	359 (10)
Maintaining work-life balance	151(4.2)	328(9.2)	512(14.3)	1404(39.2)	789(22)	396(11.1)
Management and Teamwork principles	140(3.9)	295(8.2)	566(15.8)	1574(44)	709(19.8)	296(8.3)
Physicians’ social responsibility in accidents and disasters	141(3.9)	320(8.9)	655(18.3)	1501(41.9)	684(19.1)	279(7.8)
Religious law in medical practice	366(10.2)	348(9.7)	547(15.3)	1412(39.4)	639(17.8)	268(7.5)
Health system structure	130(3.6)	301(8.4)	655(18.3)	1640(45.8)	604(16.9)	250(7)
National Laws of medicine (medical system regulations, medical judicial laws, Etc.)	199(5.6)	498(13.9)	730(20.4)	1453(40.6)	479(13.4)	221(6.2)
Philosophy of Medicine	380(10.6)	490(13.7)	789(22)	1234(34.5)	467(13)	220(6.1)
Role of logic and mathematics in clinical decision-making	455(12.7)	492(13.7)	766(21.4)	1187(33.2)	445(12.4)	235(6.6)
Social prescribing^1^	644(18)	495(13.8)	822(23)	1001(28)	452(12.6)	166(4.6)

**Table 3:** Importance of having knowledge about interdisciplinary items from the point of view of all graduated medical physicians and senior medical students (percentage in parentheses)

Interdisciplinary Item	None	Very Little	Little	Medium	High	Very High
**Self-anger management**	34(0.9)	51(1.4)	36(1)	297(8.3)	1110(31)	2052(57.3)
**Maintaining work-life balance**	40(1.1)	69(1.9)	85(2.4)	261(7.3)	796(22.2)	2329 (65.1)
**Social determinants of health**	24 (0.7)	27 (0.8)	53 (1.5)	402 (11.2)	1216(34)	1858 (51.9)
**National laws of medicine (medical system regulations, medical judicial laws, etc.)**	42(1.2)	76(2.1)	88(2.5)	306(8.5)	868(24.2)	2200(61.5)
**Physicians’ social responsibility in accidents and disasters**	37(1)	46(1.3)	70(2)	413(11.5)	1391(38.9)	1623(45.3)
**Social and economic consequences of disease**	27(0.8)	37(1.0)	67 (1.9)	439 (12.3)	1378(38.5)	1632 (45.6)
**Management and teamwork principles**	46(1.3)	74(2.1)	75(2.1)	537(15)	1325(37)	1523(42.5)
**Role of gender, racial, ethnic, social, economic, and political status of the patients in providing services to them**	82(2.3)	51(1.4)	131(3.7)	620(17.3)	1280 (35.8)	1416(39.6)
**Social prescribing**	66(1.8)	71(2)	143(4)	742(20.7)	1400(39.1)	1158 (32.3)
**Health system structure**	59(1.6)	91(2.5)	131(3.7)	770(21.5)	1323(37)	1206(33.7)
**Philosophy of medicine**	139(3.9)	162(4.5)	320(8.9)	1050(29.3)	1022(28.5)	887 (24.8)
**Role of logic and mathematics in clinical decision-making**	147(4.1)	198(5.5)	404(11.3)	1238(34.6)	967(27)	626(17.5)
**Religious law in medical practice**	444(12.4)	281(7.8)	448(12.5)	918(25.6)	807(22.5)	682(19.1)

**Table 4:** Educational need assessment about interdisciplinary items from the point of view of all studied groups (percentage in parentheses)

Interdisciplinary Items	None	Very Little	Little	Medium	High	Very High
**National laws of medicine (medical system regulations, medical judicial laws, etc.)**	99(2.8)	41(1.1)	78(2.2)	584(16.3)	855(23.9)	1923(53.7)
**Maintaining work-life balance**	109(3)	67(1.9)	76(2.1)	627(17.5)	743(20.8)	1958(54.7)
**Self-anger management**	138(3.9)	76(2.1)	103(2.9)	614(17.2)	815(22.8)	1834(51.2)
**Social determinants of health**	124(3.5)	61(1.7)	104(2.9)	777(21.7)	1099(30.7)	1415(39.5)
**Physicians’ social responsibility in accidents and disasters**	129(3.6)	61(1.7)	95(2.7)	787(22)	1022(28.5)	1486(41.5)
**Social and economic consequences of disease**	125(3.5)	65(1.8)	103(2.9)	790(22.1)	1098(30.7)	1399(39.1)
**Social prescribing**	148(4.1)	64(1.8)	110(3.1)	781(21.8)	948(26.5)	1529(42.7)
**Management and teamwork principles**	143(4)	77(2.2)	119(3.3)	841(23.5)	970(27.1)	1430(39.9)
**The role of the science of logic and mathematics in the principles of clinical decision-making and the processes of providing services by doctors to patients**	224(6.3)	129(3.6)	181(5.1)	1079(30.1)	914(25.5)	1053(29.4)
**Role of gender, racial, ethnic, social, economic, and political status of the patients in providing services to them**	172(4.8)	83(2.3)	125(3.5)	926(25.9)	1026(28.7)	1248(34.9)
**Health system structure**	171(4.8)	98(2.7)	143(4)	942(26.3)	991(27.7)	1235(34.5)
**Philosophy of medicine**	270(7.5)	142(4)	183(5.1)	1090(30.4)	855(23.9)	1040(29.1)
**Religious law in medical practice**	703(19.6)	192(5.4)	218(6.1)	932(26)	667(18.6)	868(24.2)

**Table 5:** Educational needs about interdisciplinary items from the point of view of studied subgroups; presented as number (%) of high to very highly/ number (%) of none to moderate

Studied Subgroups/Expressed Educational Needs	Faculty members Sub-specialists and Specialists (*n* = 778)	Non-faculty members Sub-specialists and Specialists (*n* = 730)	General Practitioners (*n* = 1388)	Senior Medical Students (*n* = 684)	*P* Value
**National laws of medicine (medical system regulations, medical judicial laws, etc.)**	578 (74.3)/200(25.7)	559 (76.6)/171(23.4)	1090 (78.5) /298(21.5)	551 (80.6)/133(19.4)	0.024
**Maintaining work-life balance**	580 (74.6)/198(25.4)	537 (73.6)/193(26.4)	1046 (75.4)/342(24.6)	538 (78.7)/146(21.3)	0.136
**Self-anger management**	567 (72.9)/211(27.1)	532 (72.9)/198(27.1)	1049 (75.6)/339(24.4)	501 (73.2)/183(26.8)	0.395
**Social determinants of health**	556 (71.5)/222(28.5)	497(68.1)/233(31.9)	1001(72.1)/387(27.9)	460 (67.3)/224(32.7)	0.059
**Physicians’ social responsibility in accidents and disasters**	552 (70.1)/226(29)	483(66.2)/247(33.8)	1003 (72.3)/385(27.7)	470 (68.7)/214(31.3)	0.025
**Social and economic consequences of disease**	543 (69.8)/235(30.2)	492(67.4)/238(32.6)	983 (70.8)/405(29.2)	479 (70)/205(30)	0.441
**Social prescribing**	545 (70.1)/233(29.9)	461(63.2)/269(36.8)	989 (71.3)/399(28.7)	482(70.5)/202(29.5)	0.001
**Management and teamwork principles**	521 (67)/257(33)	470 (64.4)/260(35.6)	935 (67.4)/453(32.6)	474 (69.3)/210(30.7)	0.264
**The role of the science of logic and mathematics in the principles of clinical decision-making and the processes of providing services by doctors to patients**	435 (55.9)/343(44.1)	416 (57)/314(43)	768 (55.3)/620(44.7)	348 (50.9)/336(49.1)	0.103
**Role of gender, racial, ethnic, social, economic, and political status of the patients**	495 (63.6)/ 283(36.4)	452 (61.9)/278(38.1)	898 (64.7)/490(35.3)	429 (62.7)/255(37.3)	0.608
**Health system structure**	467 (60)/311(40)	445 (61)/285(39)	873 (62.9)/515(37.1)	441 (64.5)/243(35.5)	0.28
**Philosophy of medicine**	432 (55.5)/346(44.5)	389 (53.3)/341(46.7)	740 (53.3)/648(46.7)	334 (48.8)/350(51.2)	0.077
**Religious law in medical practice**	347 (44.6)/431(55.4)	293 (40.1)/437(59.9)	577 (41.6)/811(58.4)	318 (46.5)/366(53.5)	0.05

## Discussion

The primary objective of this study was to evaluate the necessity of incorporating interdisciplinary subjects into Iran’s existing medical education curriculum. This study found that in overall, medical graduates and students’ self-assessed interdisciplinary knowledge ranged from low to moderate and mostly stated that they never had been educated before about interdisciplinary subjects. Furthermore, topics like self-anger management, maintaining work-life balance, and understanding social determinants of health were identified as the most crucial educational needs. In contrast, subjects like sharia law in medical practice, philosophy of medicine and the role of logic and mathematics in clinical decision-making were deemed less significant by the respondents. Educational needs varied also among studied subgroups; faculty members prioritized work-life balance, whereas non-faculty specialists, general practitioners, and senior students were more concerned with education about national medical laws.

Our findings are broadly congruent with existing literature, although some points of divergence are noteworthy. For example, our results align with other studies in advocating for interprofessional learning activities as a means of curriculum integration [4, 16, 17]. A study defined that the core competencies for interprofessional education can be summarized into five themes including: roles and responsibilities, ethical practice, conflict resolution, communication and collaboration and teamwork [4]. These items were also among demanded topics that we found in our study. Further, integrating socio-ecological determinants of health, as corroborated by Rao R, et al. offers a multidimensional perspective on health [18]Interdisciplinary approach to the health, encourages medical students to account for various social, economic, and environmental factors that can influence health outcomes [19]. Importance of work-life balance and prevention of burn-out of health-care workers and medical students, which demanded by participants in this study, were also emphasized by other studies [20, 21].

Another avenue which was demanded by at least one half of the participants in this study for medical curriculum enrichment, was developing a more dynamic education of ethics and law in medicine. This approach is needed for building appropriate rapport between medical doctors and their patients meanwhile understanding of the legal complexities which are inherent in medical practice, as also another study concluded [22]. Our study also found that lack of empowerment of medical students in anger management is another existing gap in the current medical curriculum, as also resulted by another study [23]. Nevertheless, some of our findings are in tension with other studies. These controversies may come from differences in the studied groups and their needed priorities, contents of medical curriculums, socio-economic-legal contexts, health systems structures, policies or methodologies of studies. For example, in opposite to our findings, a survey about the current state of evidence-based medicine (EBM) curricula in 115 US and Canadian medical schools, concluded that the philosophy of medicine is one the most critical aspects of medical humanities that can be integrated into the medical curriculum [7]. Participants in our study did not give a high demanded score for this topic.

In Iran, there is a centralized educational system, which is conducted similarly in all regions regardless of different educational priorities and heterogenous contexts. Importance of context in the medical education was shown in some studies [24]. In other word, the concept of one-size-fits-all in designing medical education should be avoided [25]. In this study, we also found a low to intermediate level of knowledge about interdisciplinary topics among participants. Carden et al., discussed about how a lack of self-awareness can lead to a disconnect between how a person perceives themselves and how others perceive them [26]. The next issue which was assessed in this study, was the stage of medical education in which interdisciplinary topics were taught. More than half of the participants stated that they had not received any interdisciplinary training. This shows that IE has not been well implemented, and the medical education curriculum in Iran requires serious revision. This finding is on par with a previously conducted qualitative study in Iran [27]. Prioritization is also very important in adding interdisciplinary subjects to the medical education curriculum. For this reason, participants in this study were asked to declare their educational needs priority for each subject. Different priorities were presented by studied subgroups. In line to our findings, another study showed that factors that influence interdisciplinary learning are personal epistemics, individual learning preferences, and the synergy that is achieved throughout interdisciplinary learning. A study concluded that organizing interaction among different disciplines could make them aware of inequalities, implicated biases and assigned status of different groups. These empirical results are pivotal to tailor interdisciplinary education to each specific discipline and to take interdisciplinary learning to a higher level of maturity [28]. For interdisciplinary education to have effective results, it must be accepted at all levels. chief directors, especially in colleges and hospitals, should move their institutional vision toward interdisciplinary cooperation as the key to achieving better outcomes in education and treatment. This should be done by providing resources and incentives for interdisciplinary collaborations and creating a solid cultural expectation of interdisciplinary cooperation [29, 30]. Acquaintance of the medical doctors with management approaches, organizational and professional behaviors and teamwork principles, was demanded by the majority of participants in our study. A study emphasized the need for IE integration into the medical education curriculum to improve the quality of medical services, education, and treatment outcomes. The solution to the current problems lies in enriching existing courses with interdisciplinary topics rather than establishing new fields of study or courses [31]. Regarding the timing of integration, the optimal timing for introducing interprofessional Education (IPE) into medical curricula remains a subject of ongoing debate [32]. While a study suggests that incorporating IPE during the clinical years could be beneficial for fostering professional identities and encouraging interdisciplinary collaboration, it also cautions that late introduction may conflict with students’ growing focus on specialized clinical skills [33]. Conversely, the early introduction of IPE in pre-registration healthcare courses has been shown to be effective in dispelling negative attitudes and avoiding the perpetuation of stereotypes [34–36]. A systematic review found inconclusive evidence on the ideal timing for IPE, noting that both pre-clinical and clinical interventions have demonstrated some level of success, with later interventions often yielding longer-lasting and statistically significant impacts [37]. Given these complexities, a prudent approach may involve initiating IPE early in the curriculum and maintaining it throughout, although additional well-designed studies are needed to clarify this educational quandary further. Another study remarked that to perform the initial interventions on an experimental basis, it is possible to provide the theoretical familiarity of medical students with these subjects in the pre-clinical years and courses such as public health, social medicine, the etiquette of medicine and forensic medicine [36]. At clinical levels, the students’ practical familiarity in these areas should also be considered [38]. On the other hand, medical graduates should be required to learn the topics above as part of retraining courses and should be given some privileges regarding retraining courses. Therefore, drawing a roadmap and developing a strategic and operational educational plan before these interventions seem to be mandatory [39]. However, conducting a comprehensive intervention for IE inclusion within the medical curriculum faces several obstacles. One significant hurdle is the absence of a unified communication channel for faculty members and students, which complicates the collection of reliable and accurate data [40–42].

### Limitations and recommendations

Despite a rigorously methodology and mixed pattern of study, this study had some limitations and did not include all things (such as how and when enrichment of medical curriculum should be implemented), that are should be known about inclusion of IE subjects into the medical curriculum. Also, our digital approach in quantitative data gathering, may have lost older ages who were less or non-computer user. As a recommendation, future studies should investigate current medical curriculum regarding those lessons which can be enriched or replaced by IE items, meanwhile preventing of curriculum fattening. Furthermore, acquisition of medical education experts’ opinion about how and when IE topics should be educated and evaluated, is also important to be defined in the next researches. Knowing about human sciences experts’ views about enrichment of IE in the medical education is also important. Considering characteristics of different contexts, cultures and socio-economic backgrounds of people in different parts of country is also necessary for planning effective interventions. Therefore, an action plan which includes all steps of need assessment, planning, implementation and evaluation should be made as the road map of these activities and future interventions.

**In conclusion**, this study revealed a low to moderate level of knowledge about interdisciplinary topics among both graduated medical physicians and senior medical students. These groups showed a strong demand and tendency to know and to be educated about these topics. These findings underscore the urgency for educational reforms to meet the interdisciplinary needs of medical professionals in Iran. Taking advantage of the experiences of countries that have taken successful steps in the field of integration of interdisciplinary sciences in medical education can pave the way for making the necessary reforms in this regard in Iran, although differences in the contexts and the influence of various factors should not be kept out of the sight.

## Data Availability

The datasets generated and/or analyzed during the current study are not publicly available due to sensitive confidential issues but are available from the corresponding author on reasonable request.

## Footnotes

1 Social prescribing tries to provide non-medical and non-pharmacological services to create social support for patients and a better care of their health. (e.g., nature tours, art programs, stress and anger management classes, job creation, etc.).
